# 

**Published:** 1898-09

**Authors:** 


### CONTENTS.

| | PAGE |
|---|---|
| ORIGINAL ARTICLES: | |
| President's Address. By D. E. SALMON, D.V.M. | 583 |
| Veterinary Notes from Europe. By LEONARD PEARSON, B.S., V.M.D. | 592 |
| State Control of Hog-cholera. By M. H. REYNOLDS, D V.M. | 601 |
| Useful Points of Value in a Country Practice. By S. S. WHITBECK, D.V.M | 614 |
| A New Treatment of Parturient Paresis of Cows. By OLOF SCHWARZKOPF, V.M. | 619 |
| Wild and Cattle Diseases. By H. D. FENNIMORE, D.V.S. | 625 |
| SELECTIONS: | |
| To Detect Horseflesh from Beef | 627 |
| Great Trees | 627 |
| Poisoning by Caterpillars | 627 |
| THERAPEUTIC NOTES: | |
| Reduction in Price of Antipyrin | 628 |
| Scabinol for Dog-mange | 628 |
| Toxin of Tuberculosis Bacillus | 628 |
| Quinine for Hypodermatic Use | 628 |
| A New Counter-irritant | 629 |
| Ointment for Itching Skin Diseases | 629 |
| A New Antiseptic | 629 |
| Carbolic Salve Containing Calomel | 629 |
| Decolorized Tincture of Iodine | 629 |
| Antipyrine in Ophthalmia | 629 |
| Antiseptic Ointment | 629 |
| Wine of Cascara Sagrada | 630 |
| Lassar's Paste for Eczema | 630 |
| Contagious Cerebro-spinal Meningitis | 630 |
| Phosphoric Acid for Foul Ulcers | 630 |
| Antiseptic Surgical Dressing | 630 |
| Local Anæsthetics | 630 |
| Exciting Cause of Hydrophobia | 631 |
| Powder for Wounds | 631 |
| Solvent Action of Boric Acid | 631 |
| Hall's Solution of Strychnine | 631 |
| Barbadoes Aloes Not in the Market | 631 |
| REPORTS OF CASES: | |
| Arytenoideraphy—a New Surgical Treatment for Roaring. By L. A. MERILLAT, V.S. | 632 |
| Stomach-staggers in Horses. By H. W. SMITH, V.S. | 633 |
| EDITORIALS | 635 |
| NECROLOGY | 636 |
| AT OMAHA | 637 |

LIBRARY

SURGEON GENERAL'S OFFICE

